# ICF-Based Job Performance Predictors for South Korean Industrial Accident Workers: Population-Based 3-Year Longitudinal Analysis

**DOI:** 10.3390/ijerph19137822

**Published:** 2022-06-25

**Authors:** Gayeong Eom, Seonjae Been, Haewon Byeon

**Affiliations:** 1 Department of Statistics, Graduate School, Inje University, Gimhae 50834, Korea; djarkdud2@naver.com; 2 Department of Digital Anti-Aging Healthcare (BK21), Graduate School, Inje University, Gimhae 50834, Korea; beensj986@gmail.com

**Keywords:** latent growth modeling, ICF, job performance, industrial accident workers

## Abstract

Since workers who have experienced industrial accidents may have a physical impairment, their workload is very likely to be less than before the industrial accident. This epidemiological study identified ICF-based predictive factors affecting the work performance of South Korean workers who were economically active after undergoing medical treatment (rehabilitation) related to injuries caused by industrial accidents by using the Panel Study of Worker’s Compensation Insurance (2018–2020) as a reference. We analyzed 1383 subjects who were engaged in economic activities. The explanatory variables included participation factors, activity factors, personal factors, physical function factors, and rehabilitation service factors. The outcome variables were defined by subjective evaluations of current job performance (0 and 10 points). This study analyzed the trajectory of change in work performance and change function predictors over time by using latent growth modeling (LGM). This study found mental activity, indoor activity, occupational activity, disability grade, socioeconomic status, the length of recuperation, self-esteem, and self-efficacy as significant predictors. The results of this study suggested that it would be necessary to prepare a systematic program that considers activity factors to support the independent daily life activities and social participation of workers injured by industrial accidents.

## 1. Introduction

Industrial accidents affect economics at various levels, including countries, companies, and individuals. At the national level, it influences the economic loss of the entire country due to direct loss estimation (e.g., insurance benefits) and indirect loss estimation [1]. At the company level, it includes effects due to disruption in production and damaged corporate image [2]. At the individual level, it means physical and mental pain and a threat to an individual’s livelihood [3]. The 2020 industrial accident status (the Ministry of Employment and Labor) showed that insurance benefits (direct loss) were 4651 million USD and the estimated economic losses, including direct and indirect losses, were 23,254 million USD [4]. This is an increase of 8.45% from the previous year (2019) [4].

In particular, the estimation of economic losses has been steadily increasing from 2016 to the present. It was also confirmed that both the estimation of economic losses and lost workdays increased considerably in 2018. Therefore, identifying the work performance capabilities and related factors of workers injured by industrial accidents was an important public health issue. The press release of the Ministry of Employment and Labor and the Korea Labor Institute suggested that it was caused by the expansion of workplaces covered by the Industrial Accident Compensation Insurance, the increased number of applications due to the recognition of an accident while commuting as an industrial accident, re-application due to the revised Notice of Cerebrovascular Disease, and most importantly, the simplification of the industrial accident application process due to the abolition of the employer (the policyholder of the industrial accident compensation insurance) verification system [5,6].

Industrial accidents affect companies in ways such as production disruption and damaged corporate image. Previous studies [2] also showed that it significantly influenced the business performance, such as regarding the sales growth rate and operating profit, which could lead to social and economic losses. Especially, it may cause a loss of skilled human capital [1,7]. Furthermore, the loss of human capital is not only a big loss at the corporate level but also a huge loss at the individual level of the workers [8]. Since workers who experience industrial accidents cannot accept the resulting disability psychologically, they become obsessed with physical or mental disabilities, which makes them view themselves negatively [9]. Moreover, they experience greater psychological difficulties due to physical and environmental changes, which may result in maladaptation to social life [9,10]. In other words, workers are more likely to lose their workforce and be unable to continue their occupational activities due to an industrial accident temporarily or permanently.

A worker’s return to work is an important factor in terms of social and economic aspects [11], employment stability, and quality of life [12,13]. Therefore, it is necessary to recover the workforce more than anything for workers injured by industrial accidents to continue their occupational activities [14]. Job performance is used as a concept to measure the recovery of the workforce [14]. Choi & Lee, 2015 [15] defined job performance as the knowledge, skills, attitudes, behaviors, and understanding of the job performance process required to perform the job. In other words, good job performance means that a worker has good work performance [16], and one with excellent work performance may return to work relatively easier. The Korea Workers’ Compensation & Welfare Service [17] reported that 95.7% of employers at workplaces where an industrial accident occurred responded that they would positively consider the return of workers injured by industrial accident to work if specific medical information (e.g., possible to return to work) is provided to the employer.

In summary, it is necessary to introduce a new evaluation system that can examine the impact of physical disability on the work of workers injured by an industrial accident instead of the existing disability evaluation system that simply reflects the physical disability in determining the disability of workers injured by industrial accidents. The International Classification of Functioning, Disability, and Health (ICF) can be used as a tool to properly conduct such an assessment.

The ICF was first introduced in South Korea in 2018 [18]. It introduced a new concept of disability that combined social and physical environmental factors rather than medical or biological disabilities. It is provided as a tool to classify a wide range of health-related information. Since the ICF was useful for recording a profile related to the functional performance, disability, and health of an individual in various sectors, including health and medical care, disability, and social welfare because it includes health and environmental factors, factors affecting the work performance of workers injured by industrial accidents could be classified into ICF codes and provided [19]. This epidemiological study identified ICF-based predictive factors affecting the work performance of South Korean workers who were economically active after completing medical treatment (rehabilitation) related to injuries caused by industrial accidents by using the Panel Study of Worker’s Compensation Insurance (2018–2020), which could represent South Korean workers who were injured by industrial accidents.

## 2. Theoretical Background

### 2.1. ICF

**Figure 1:**
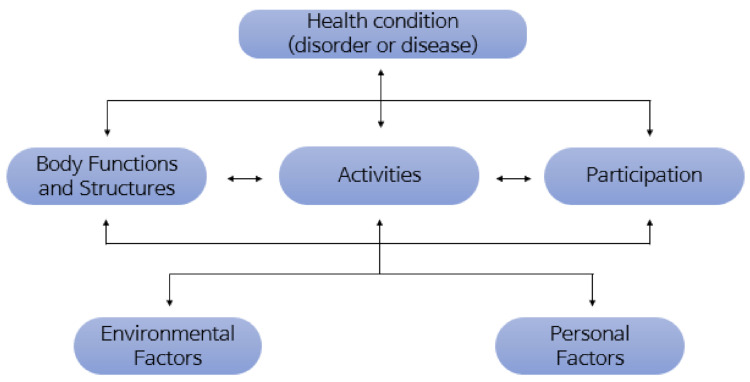
Concept of the ICF.

Disability, as defined by the ICF, is a health state that encompasses interactions between the body, environment, and individuals [19] (Figure 1). Therefore, the ICF-based evaluation has the advantage of being able to identify the functional limitations of the environmental and social aspects of the disabled in their daily life clearly and efficiently [20].

After the ICF was introduced in South Korea, many previous studies evaluated the ICF in various academic fields. Seoul National University Bundang Hospital, 2012 [21] conducted studies on the development of a disability management program for cancer survivors and used the ICF to develop a comprehensive functional evaluation tool that could reflect various aspects to provide rehabilitation services. Lee et al., 2018 [22] also used the ICF in an intervention plan for stroke patients by examining the relationship with mobility items among the tools to measure gait speed and mobility and the details items of the ICF. Other studies related to industrial accidents also used the ICF. Lee & Kim, 2018 [23] identified factors influencing the job performance of female workers and applied factors related to job return to the ICF framework.

These previous studies [21,22,23] selected the degree of others’ help, difficulty in the activities of daily living (ADL), difficulty in the instrumental activities of daily living (IADL), and job performance as the major factors of the ICF in ICF activity factors and participation factors. This study was different from previous studies, which did not use the ICF, in that it coded the difficulty in the ADL and the difficulty in the IADL as d2301 (managing the activities in daily life) and d2302 (completing the activities in daily life).

### 2.2. Job Performance and Return to Work

Workers injured by industrial accidents express psychological difficulties, aftereffects, and physical difficulties in performing their jobs [16]. As a result, workers injured by industrial accidents may have lower performance compared to their previous work performance and experience difficulties in returning to work and maintaining their work. Two of the purposes of industrial accident compensation insurance are to stably reproduce the labor force s by preventing labor loss and reducing the threat to the living of workers injured by industrial accidents and their family members [24]. In other words, helping workers injured by industrial accidents return to work as soon as possible positively influences employers, workers, workers’ families, and communities. Bae, 2017 [14] evaluated the labor market transition of workers injured by industrial accidents and used the subjective evaluation of work performance as a factor related to whether workers injured by industrial accidents returned to work. Bae, 2017 [14] reported that better subjective evaluation of one’s work performance significantly increased the prevalence of a return to work.

Nevertheless, previous studies [25,26,27] just evaluated the social support, daily living performance, satisfaction, and quality of life of workers injured by industrial accidents or persons with disabilities due to industrial accidents. There are not enough epidemiological studies that analyze the factors affecting the work performance of workers injured by industrial accidents after returning to work.

### 2.3. Factors for Predicting Work Performance

There were not enough domestic studies focusing on job performance. Therefore, not many previous studies analyzed the factors affecting job performance. The following are factors identified by some previous studies. Kim et al., 2016 [16] showed that job performance decreased when the subject was older, had a lower educational level, had poorer health, and was divorced. Among job-related factors, the length of recuperation and the level of disability had an impact on the decrease in work performance.

Moreover, those with a disability of grades between 1 and 7 had lower job performance than those who answered that they had no disabilities. Lee & Kim, 2018 [23] reported that personal factors (e.g., educational level, workplace, disability grade, and the presence of disability), environmental factors (e.g., economic activities, employment status, employment period, socioeconomic status, the length of recuperation, and work performance), and participation factors (e.g., the degree of others’ help and difficulties in IADL) significantly affected job performance. This study selected explanatory variables by referring to the results of these previous studies [16,23].

## 3. Materials and Methods

### 3.1. Research Data and Research Subjects

This was a secondary-data use study that analyzed the Panel Study of Worker’s Compensation Insurance (PSWCI) surveyed by the Korea Workers’ Compensation and Welfare Service. The PSWCI is the follow-up data of workers injured by industrial accidents from the time of the industrial accident to three years after the end of recuperation. It was conducted to understand the changes in the lives of workers injured by industrial accidents. The PSWCI received research ethics approval from Statistics Korea (no-0439001), and the population was 75,392 workers who completed occupational accident recuperation in South Korea as of 2017. This study sampled subjects using the stratified systematic sampling method by using stratification variables (disability grade, gender, and age), and 3924 subjects were finally selected for the survey. This study used a 1:1 interview survey using a tablet PC targeting workers who completed occupational accident recuperation from August 2018 to October 2020. Please refer to Jung & Shin, 2021 [28] for a detailed description of the data source.

This study excluded 1911 people who were not currently working after discharge among 3294 workers who participated in all rounds of the PSWCI from the 1st year (2018) to the 3rd year (2020). Finally, this study analyzed 1383 subjects who were engaged in economic activities (e.g., those who returned to their original work, those who were re-employed, and those who were self-employed).

### 3.2. Variable Selection and Detailed ICF Classification

Table 1 shows the application of the ICF classification system according to the variables. The ICF classification categorized functional performance and disability factors into physical factors, activities, and participation and classified background factors into four detailed factors by dividing them into environmental factors and personal factors. After selecting variables related to each factor, it was coded with ICF codes. Among physical elements, self-esteem and self-efficacy were used as variables. For activities and participation, difficulties in daily life, participation in meetings, and satisfaction with family, relatives, and social relationships were used as variables. Vocational and social rehabilitation services were used as variables for environmental factors. Personal factors included characteristics created by industrial accidents (e.g., length of recuperation, the presence of disability, and disability grade), types of industrial accidents, and socioeconomic status, which were identified as influencing factors in previous studies.

### 3.3. Measurement and Definition of Variables

The outcome variables were defined by subjective evaluations of current job performance. The subjective evaluation of job performance was a self-reporting method, graded between 0 and 10 points: 0 points means the complete loss of work performance, and 10 points refer to the complete recovery of work performance.

The explanatory variables included participation factors, activity factors, personal factors, physical function factors, and rehabilitation service factors. Among the participation factors, the frequency of meeting participation was defined as the mean of three items (i.e., religious meetings, social meetings, and clubs), which were on a 6-point scale (1 = never participated, 2 = less than once a month, 3 = once a month, 4 = two or three times a month, 5 = once a week, and 6 = two or more times a week). Satisfaction with daily life (i.e., family relationships, relative relationships, and social relationships) was measured by a 5-point scale: “very satisfied (1 point)” to “very dissatisfied (5 points)”. It was defined as the level of satisfaction through inversing the score.

Among the activity factors, mental activity, indoor activity, outdoor activity, and occupational activity were on a 5-point scale, ranging from “feel very difficult (1 point)” to “doesn’t feel difficult at all (5 points)”. Personal factors included disability grade (1 = between 1 and 3 grade, 2 = between 4 and 7 grade, 3 = between 8 and 9 grade, 4 = between 10 and 12 grade, 5 = between 13 and 14 grade, or 6 = no disability), industrial accident types (1 = occupational accident, 2 = occupational illness, or 3 = commuting accident), socioeconomic status (1 = upper class, 2 = upper-middle class, 3 = lower-middle class, or 4 = lower class), and the length of recuperation (1 = 3 months or less, 2 => 3months and ≤6 months, 3 => 6 months and ≤9 months, 4 => 9 months and ≤1 year, 5 => 1 year and ≤2 years, or 6 => 2 years). In this study, it was interpreted that a higher disability grade was closer to no disability, and a lower disability grade indicated a greater degree of physical injury.

In the physical function factors, self-efficacy was defined as the mean of 23 items on a 5-point scale: from “not at all (1 point)” to “strongly agree (5 points)”. Cronbach’s alpha of self-efficacy was 0.907, showing very high reliability. Self-esteem was defined as the mean of ten items on a 4-point scale: “mostly not (1 point)” and “mostly so (4 points)”. Cronbach’s alpha of self-esteem showed a reliability of 0.618. Rehabilitation service factors consisted of the utilization of vocational rehabilitation services (0 = not used, or 1 = used) and the utilization of social rehabilitation services (0 = not used, or 1 = used). Table 2 presents the questionnaires for measuring the variables.

### 3.4. Development of Latent Growth Model

This study analyzed the trajectory of change in work performance and change function predictors over time by using latent growth modeling (LGM). LGM is known as a suitable method for longitudinal studies that focus on changes in the job performance of workers injured by industrial accidents and predictive factors over time, as in this study, because it presents a pattern of change by identifying the changes in individuals and groups over time based on longitudinal data [29].

LGM conducts analysis in two major stages. In the first stage, an unconditional model is applied. The unconditional model calculates the intercept and slope of the mean developmental curve by fitting the repeated measures of each individual after measuring the developmental curve (the change in job performance over time) from the 1st to 3rd period. The second stage conducts conditional model analysis, and it validates the conditional model by linking the intercept and slope of latent factors obtained in the first stage with various predictive factors. It analyzed changes over time by factors before verifying the trajectory of change between variables through LGM. When the change increased or decreased constantly and linearly at each point, a linear growth model was selected. If no change was observed at each time point, a no-growth model was selected. The fit of the model was determined using χ², root mean square error of approximation (RMSEA: an absolute fit index), and Tucker–Lewis index (TLI: an incremental fit index), and comparative fit index (CFI). This study defined that a model had a good fit when the RMSEA was 0.08 or less, SRMR was 0.06 or less, and TLI and CFI were 0.90 or more based on the results of previous studies [30,31,32].

## 4. Results

### 4.1. General Characteristics of Subjects

Table 3 shows the demographic characteristics based on the subjects’ responses in the first year (baseline). There were more males (1183 subjects, 85.5%) than females (200 subjects, 14.5%). Subjects in their 50s were the most abundant (521 subjects, 37.7%). In terms of socioeconomic status, 93% of subjects perceived that they belonged to a lower-middle class (869 subjects, 62.8%) or a lower class (417 subjects, 30.2%). In terms of education level, high school graduation was the most common (693 subjects, 50.1%).

Table 4 shows the results of industrial accident-related frequency analysis. In terms of industrial accident types, the occupational accident accounted for 1311 subjects (94.8%). The most common length of recuperation was >3 months and ≤6 months (571 subjects, 41.3%). As for the frequency of service use, 110 subjects (8.0%) used vocational rehabilitation services, and 195 subjects (14.1%) received social rehabilitation services.

### 4.2. Descriptive Statistics of Predictors

Table 5 presents the descriptive statistics of the predictive model’s predictors. Each factor was considered to satisfy the normal distribution condition when the skewness was less than 2 and the kurtosis was less than 4 [33]. Although most factors of this model met the normal distribution requirements, industrial accident type, vocational rehabilitation service use, and social rehabilitation service use did not satisfy them. Since the data were longitudinal, this study identified the trajectory of each factor’s mean values for each time interval.

### 4.3. Types of Changes in Job Performance

**Figure 2:**
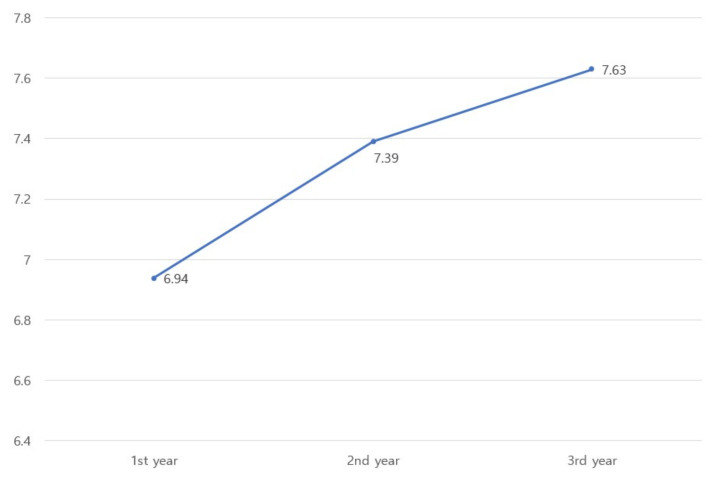
The change in the subject’s job performance over time (mean).

Figure 2 presents the change in the subject’s job performance over time. The data confirmed changes in job performance over time (mean = 6.94 in the 1st year, mean = 7.39 in the 2nd year, and mean = 7.63 in the 3rd year). Therefore, this study selected unconditional LGM in the first-stage analysis.

**Figure 3:**
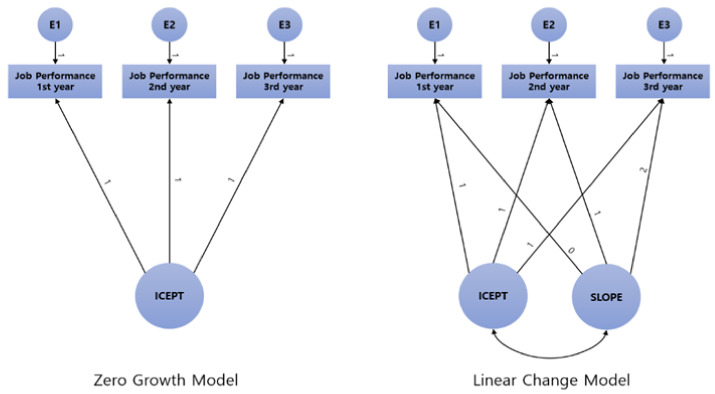
Schematic diagrams of the zero-change model and the linear change model.

In the second stage analysis, the fit was compared and validated by analyzing the zero-growth model and the linear function model (Table 6). When testing two models (zero growth model and linear change model) for the changes in job performance over time, the TLI and CFI of the linear change model were 0.966, and the SRMR was 0.025, indicating a good fit. Therefore, this study defined the change in job performance by using the linear change model. The schematic diagrams of the zero-growth model and the linear change model are presented in Figure 3.

### 4.4. Study Model Validation

#### 4.4.1. Model Fitness

**Figure 4:**
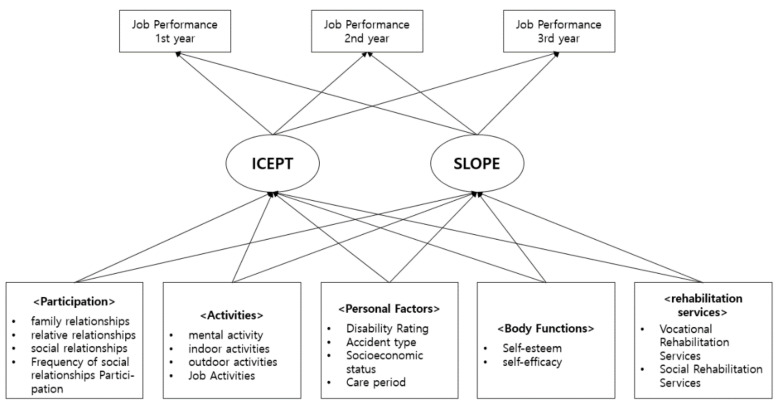
Final study models.

Figure 4 presents the final model (linear change model) on the work performance of workers injured by industrial accidents. From the initial value (first year), the coefficients of job performance in the second and third years were 1, and the coefficients of job performance in the primary slope were fixed at 0, 1, and 2, respectively. The fitness of the final study model is presented in Table 7. The value of $\chi^{2}$ (df = 19) was 122.292 (*p* < 0.001), the SRMR was 0.006, and the TLI and CFI incremental fit indices were 0.902 and 0.989, respectively. In addition, RMSEA, an absolute fit index, showed a good fit (0.063), which confirmed that the fit of the final model was good.

#### 4.4.2. Path Coefficient of the Model

Table 8 shows the results of the final model (path coefficients between variables) that analyzed the effects of explanatory variables on the intercept and slope of job performance for each factor.

Mental activity, indoor activity, and occupational activity, input variables as activity factors, had a significant impact on job performance (*p* < 0.05). However, mental activity significantly affected the intercept of job performance, but it did not influence the slope significantly. Indoor activity and vocational activity significantly influenced both intercept and slope (*p* < 0.05), which indicated that the initial value of job performance was higher when experiencing less difficulty in performing indoor and vocational activities, and the impact on job performance decreased over time.

The intercepts and slopes of disability grade, socioeconomic status, and length of recuperation among personal factors significantly affected job performance (*p* < 0.05). Although it was confirmed that a higher disability grade influenced job performance more persistently, the intercept value decreased when the socioeconomic status was lower and the length of recuperation was longer. The improvement speed of work performance increased over time.

Among physical function factors, self-esteem had a significant impact on interception (*p* < 0.05), and self-efficacy significantly affects slope (*p* < 0.05). It can be interpreted that workers with high self-esteem had significantly better work performance than workers with low self-esteem. On the other hand, self-efficacy did not influence the intercept significantly, but it significantly influenced the slope, which could be interpreted as that job performance gradually increased over time.

## 5. Discussion

Workers injured by industrial accidents need to show at least the same or similar level of job performance as before the accident to return to work successfully. This study confirmed the dynamics of the job performance of workers injured by industrial accidents and examined factors influencing the type of changes in job performance by applying LGM to workers previously injured by industrial accidents and currently employed after completing rehabilitation.

This study evaluated the factors of job performance by dividing them into activity, participation, physical function, rehabilitation services, and personal factors based on the ICF components. The results confirmed that mental activity influenced only intercept, but the intercept and slope of indoor activity and occupational activity significantly affected job performance. Indoor activity and occupational activity showed a higher interception of job performance when workers did not feel any difficulty in performing each activity. However, the improved speed of job performance decreased over time. It is believed that it was because the score was already high at the baseline stage when the indoor activity and occupational activity of workers injured by industrial accidents were measured after completing recuperation, which relatively decreased the increment. More longitudinal studies are required to understand the causal effects of indoor activities, occupational activities, and mental activities on job performance.

The results of this study showed that disability grade, socioeconomic status, and length of recuperation among personal factors significantly affected the intercept and slope of job performance dynamics. First, it was confirmed that lighter disability (closer to no disability) improved job performance recovery more, and Ryu & Kim [34] reported a similar result. Contrarily, the results of this study revealed that although the intercept of job performance was lower when the socioeconomic status of the worker was lower, and the length of recuperation was longer, the job performance of the worker increased over time. It could be because a longer recuperation period implied a more serious industrial accident and would delay the time of returning to work, which would result in a decrease in job performance and negatively influence the intercept of job performance due to fear of adaptation [35,36].

Another finding of this study was that self-esteem and self-efficacy had a significant effect on work performance. The results of this study showed that workers with high self-esteem had better job performance than workers with low self-esteem, and this trend was consistently maintained during the 3-year follow-up period. In this study, the mean intercept of self-esteem was 2.8 points, which was lower than other factors. The result agreed with the results of previous studies showing that workers injured by industrial accidents had lower psychological factors such as self-esteem than general workers due to physical damage and role loss [37,38,39,40]. On the other hand, self-efficacy tended to increase gradually over time compared to self-esteem. It could be because the mean intercept of self-efficacy was 3.5, which was higher than that of self-esteem, and it resulted in a small increment. Self-awareness, such as self-esteem and self-efficacy, can act as a defensive mechanism that alleviates negative situations for workers who have experienced industrial accidents [41]. Additional cohort studies are required to understand the causal relationship between self-esteem, self-efficacy, and job performance.

In this study, vocational rehabilitation services and social rehabilitation services did not have a significant effect on job performance. The result disagreed with the results of previous studies [42,43,44], which showed that the experience of rehabilitation services positively affected employment. This disagreement could be because this epidemiologic study targeted active job seekers after recuperation, and only 8% and 14.1% of workers injured by industrial accidents used vocational rehabilitation services and social rehabilitation services, respectively. It is necessary to further prove the effects of rehabilitation services for industrial accident workers on their job performance through community-based cohort studies.

This study confirmed the physical function, social activity, and environmental factors of industrial accident workers based on the ICF code using longitudinal data that could represent South Korean workers injured by industrial accidents. This study is important because it presented baseline data for workers injured by industrial accidents to return to work by proving the causality with job performance.

This study had several limitations. First, since this longitudinal study analyzed only three years (2018–2020), it was limited in understanding detailed changes over time. Second, this study did not consider detailed characteristics of job performance because it did not classify workers’ economic activity types such as job type or employment type. Third, this study could not apply separate ICF codes for measuring the job performance of industrial accident workers. In the future, additional studies will need to identify the ICF codes required to measure the job performance of workers.

## 6. Conclusions

This longitudinal study identified the work performance factors of workers injured by industrial accidents focusing on activity, participation, physical function, rehabilitation services, and personal factors. This study found mental activity, indoor activity, occupational activity, disability grade, socioeconomic status, the length of recuperation, self-esteem, and self-efficacy as significant predictors. The results of this study suggested that it would be necessary to prepare a systematic program considering activity factors to support the independent daily life activities and social participation of workers injured by industrial accidents. Furthermore, based on the results of this study, it will be needed to provide psychological rehabilitation services from the community aspect, such as self-esteem and self-efficacy after discharge, to restore the job performance of workers injured by industrial accidents. Additional community-level cohort studies are required to prove the causality of the work performance predictors of workers injured by industrial accidents identified in this study.

## Figures and Tables

**Table 1:** The application of the ICF classification system according to variables.

Factor			Variable	ICF Code	
Functioning and Disability	Body Functions and Structures	Body Functions	Self-esteem	B126	Temperament and personality functions
			self-efficacy		
	Activities and Participation	Activities	mental activity	D159	Basic learning: Basic learning, other specified and unspecified
				D198	Applying knowledge: Learning and applying knowledge, other specified
				D160	Focusing attention
			indoor activities	D540	Dressing
				D510	Washing oneself
			outdoor activities	D620	Acquisition of goods and services
				D498	Moving around using transportation: Mobility, other specified
			Job Activities	D850	Remunerative employment
				D855	Non-remunerative employment
		Participation	Frequency of social relationships Participation	D910	Community life
				D920	Recreation and leisure
				D930	Religion and spirituality
			Satisfaction with family relationships	D760	Particular interpersonal relationships: Family relationships
			Satisfaction with relative relationships		
			Satisfaction with social relationships	D750	Particular interpersonal relationships: Informal social relationships
Contextual Factors	Environmental Factors	rehabilitation services	Vocational Rehabilitation Services	E5800	Health services, systems, and policies
			Social Rehabilitation Services		
	Personal Factors	Personal Factors	Socioeconomic status	none	
			Accident type		
			Care period		
			Disability Rating		

**Table 2:** Variable measurement items.

Variables		Items
Participation	Satisfaction with family relationships	How satisfied are you with your family relationship?
	Satisfaction with relative relationships	How satisfied are you with your relative relationship?
	Satisfaction with social relationships	How satisfied are you with your social relationships (relationship with friends and acquaintances)?
	Frequency of meeting participation	How often do you participate in religious gatherings, social gatherings, or clubs?
Activity	Mental Activity	Are you constantly experiencing difficulties (more than 6 months) in the following activities due to physical and mental restrictions? - Learning, remembering, and focusing
	Indoor Activity	Are you constantly experiencing difficulties (more than 6 months) in the following activities due to physical and mental restrictions? - Activities in the house, such as putting on clothes dressing, bathing, etc.
	Outdoor Activity	Are you constantly experiencing difficulties (more than 6 months) in the following activities due to physical and mental restrictions? - Outdoor activities such as shopping and visiting a doctor’s office
	Occupation Activity	Are you constantly experiencing difficulties (more than 6 months) in the following activities due to physical and mental restrictions? - Conducting vocational activities
Personal	Disability Grade	What is the disability grade determined by the Korea Workers’ Compensation and Welfare Service?
	Industrial Accident Types	What type of industrial accident did you finish the medical care for?
	Socioeconomic Status	Which of the following socioeconomic status do you belong to when considering income, occupation, education, and wealth?
	Length of recuperation	How long was the length of your recuperation until the end of medical care in 2017?
Physical Function	Self-esteem	I think I’m as valuable as others. I think I have a good personality. I feel like I am mostly a loser (inverse question). I can work as well as most other people. I don’t have much to brag about (inverse question). I have a positive attitude towards myself. I am generally satisfied with myself. I wish I could respect myself a little more. Sometimes I feel like I’m useless. Sometimes I think I’m a bad person.
	Self-efficacy	I can do the job as planned. I have a problem that I can’t start work right away when I need to (inverse question). Even if I do something wrong the first time, I do it until I make it happen. If I set an important goal, I can achieve it. I give up even before I finish something (inverse question). I avoid running into a challenging task (inverse question). If something looks too complicated, I don’t even try it (inverse question). When I work on something not pleasant, I complete it under any circumstances. When I have something to do, I start working on it right away. When I try to learn something new, if it doesn’t seem like I’ll succeed on the first attempt, I give up right away (inverse question). I can handle an unexpected issue well. If a new task looks too difficult, I don’t try to learn it (inverse question). Failure only makes me try harder. Sometimes I feel insecure about my ability to do something (inverse question). I am confident. I give up on things easily (inverse question). I don’t seem to have the ability to handle almost all the problems I face in life (inverse question). Making new friends is too difficult for me (inverse question). If I want to meet a friend, I visit the friend instead of waiting for the friend to come. If the person I’m interested in is a difficult person to be friends with, I give up on making friends quickly (inverse question). Even if I don’t like a person at first sight, I don’t easily stop making friends with the person. I don’t know what to do at social gatherings (inverse question). My sociability has made my current friends.
Rehabilitation Service	Vocational Rehabilitation Services	Utilization of vocational rehabilitation services
	Social Rehabilitation Services	Utilization of social rehabilitation services

**Table 3:** Demographic characteristics.

Classification		n	%	Classification		n	%
Gender	Male	1183	85.8	Marital Status	Single	199	14.4
	Female	200	14.5		Married	986	71.3
Age Group	30 years or younger	268	19.4		Separated	20	1.4
	40′s	357	2508		Divorced	142	10.3
	50′s	521	37.7		Bereaved	36	2.6
	60′s or older	237	17.1	Highest Level of Education	Uneducated	23	1.7
Current Socioeconomic Status	Upper Class	1	0.1		Elementary School	139	10.1
	Middle-upper Class	96	6.9		Middle School	210	15.2
	Middle-lower Class	869	62.8		High School	693	50.1
	Lower Class	417	30.2		College Graduation or above	318	23.0

**Table 4:** The results of industrial accident-related frequency analysis.

Classification		n	%	Classification		n	%
Disability Grade	1~3 grades	3	0.2	Length of Recuperation	≤3 months	304	22.0
	4~7 grades	63	4.6		≤6 months	571	41.3
	8~9 grades	120	8.7		≤9 months	313	22.6
	10~12 grades	399	28.9		≤1 year	93	6.7
	13~14 grades	485	35.1		≤2 year	85	6.1
	No disability	313	22.6		>2 years	17	1.2
Vocational Rehabilitation Services	Not used	1279	92.0	Industrial Accident Type	Occupational accident	1311	94.8
	used	110	8.0		Occupational illness	67	4.8
Social Rehabilitation Services	Not used	1188	85.9		Commuting accident	5	0.4
	used	195	14.1				

**Table 5:** Descriptive statistics of predictors.

Variables	1st Year				2nd Year				3rd Year			
	M	SD	Skew	Kurt	M	SD	Skew	Kurt	M	SD	Skew	Kurt
Satisfaction with family relationships	2.30	0.729	0.407	0.556	2.32	0.765	0.359	0.234	2.32	0.729	0.342	0.341
Satisfaction with relative relationships	2.45	0.668	0.071	0.152	2.50	0.693	0.057	0.073	2.50	0.677	0.000	0.278
Satisfaction with social relationships	2.42	0.628	−0.001	−0.157	2.41	0.677	0.188	0.035	2.42	0.662	0.109	0.012
Frequency of meeting participation	1.94	0.770	1.091	1.291	1.91	0.749	1.048	1.226	1.75	0.686	1.313	2.450
Mental Activity	4.50	0.745	−1.543	2.266	4.51	0.778	−1.821	3.623	4.60	0.717	−2.064	4.586
Indoor Activity	4.52	0.767	−1.690	2.610	4.57	0.731	−2.072	5.026	4.64	0.690	−2.262	5.806
Outdoor Activity	4.43	0.858	−1.535	1.788	4.52	0.775	−1.835	3.575	4.55	0.792	−2.053	4.364
Occupation Activity	3.65	1.223	−0.332	−1.218	3.91	1.123	−0.700	−0.629	4.08	1.053	−0.979	0.012
Disability Grade	4.62	1.078	−0.594	−0.042	4.61	1.075	−0.592	−0.032	4.61	1.073	−0.586	−0.039
Industrial Accident Types	1.06	0.245	4.600	22.30	1.06	0.245	4.600	22.30	1.06	0.245	4.600	22.30
Socioeconomic Status	3.23	0.566	−0.043	−0.229	3.18	0.572	−0.011	−0.212	3.16	0.569	0.005	−0.135
Length of recuperation	2.37	1.151	0.952	0.620	2.37	1.151	0.952	0.620	2.37	1.151	0.952	0.620
Self-esteem	2.800	0.327	0.090	0.157	2.861	0.307	−0.131	−0.335	2.847	0.308	−0.106	−0.193
Self-efficacy	3.504	0.452	0.432	−0.368	3.512	0.456	0.419	−0.240	3.521	0.426	0.374	−0.056
Vocational Rehabilitation Services	0.08	0.271	3.111	7.691	0.11	0.308	2.558	4.548	0.11	0.312	2.509	4.301
Social Rehabilitation Services	0.14	0.348	2.065	2.269	0.15	0.356	1.974	1.900	0.15	0.356	1.974	1.900

**Table 6:** Model fit of job performance.

Model	$x^{2}\left(df\right)$	TLI	CFI	SRMR	RMSEA (90% CI)	Intercept		Slope–First Stage	
						Mean	Variance	Mean	Variance
Zero-Growth Model	411.65(4) ***	0.891	0.855	0.017	0.272 (0.250–0.294)	7.395 ***	2.398 ***	-	-
Linear Change Model	97.491(3) ***	0.966	0.966	0.025	0.151 (0.126–0.177)	6.976 ***	2.998 ***	0.345 ***	0.270 ***

*** *p* < 0.001.

**Table 7:** The goodness of fit of the study model.

Model	$x^{2}\left(df\right)$	TLI	CFI	SRMR	RMSEA (90% CI)
Study Model	122.292(19) ***	0.902	0.989	0.006	0.063 (0.052–0.074)

*** *p* < 0.001.

**Table 8:** Path coefficients of the final model.

Factors	Models		Estimate	S.E	C.R	*p*
Participation	Family relationships	Intercept	0.067	0.086	0.781	0.435
		slope	−0.030	0.039	−0.780	0.435
	Relative relationships	Intercept	−0.027	0.104	−0.262	0.793
		slope	−0.018	0.047	−0.383	0.701
	Social relationships	Intercept	−0.028	0.104	−0.271	0.786
		slope	−0.026	0.047	−0.548	0.584
	Frequency of meeting participation	Intercept	−0.030	0.059	−0.510	0.610
		slope	0.009	0.027	0.322	0.748
Activity	Mental Activity	Intercept	−0.153	0.073	−2.083	0.037 *
		slope	0.016	0.033	0.498	0.618
	Indoor Activity	Intercept	0.262	0.094	2.789	0.005 **
		slope	−0.087	0.042	−2.068	0.039 *
	Outdoor Activity	Intercept	0.021	0.084	0.255	0.799
		slope	0.046	0.037	1.235	0.217
	Occupation Activity	Intercept	0.445	0.042	10.513	***
		slope	−0.041	0.019	−2.157	0.031 *
Personal	Disability Grade	Intercept	0.211	0.047	4.443	***
		slope	0.043	0.021	2.018	0.044 *
	Industrial Accident Types	Intercept	0.270	0.176	1.529	0.126
		slope	−0.051	0.079	−0.639	0.523
	Socioeconomic Status	Intercept	−0.514	0.081	−6.320	***
		slope	0.132	0.036	3.612	***
	Length of recuperation	Intercept	−0.226	0.044	−5.139	***
		slope	0.059	0.020	2.984	0.003 **
Physical Function	Self-esteem	Intercept	0.337	0.158	2.134	0.033 *
		slope	0.030	0.071	0.422	0.673
	Self-efficacy	Intercept	0.215	0.115	1.864	0.062
		slope	−0.123	0.052	−2.377	0.017 *
Rehabilitation Service	Vocational Rehabilitation Services	Intercept	−0.102	0.163	−0.625	0.532
		slope	0.076	0.073	1.049	0.294
	Social Rehabilitation Services	Intercept	−0.119	0.129	−0.920	0.358
		slope	0.062	0.058	1.072	0.284

* *p* < 0.05, ** *p* < 0.01, *** *p* < 0.001.
